# Progress in Medicine

**Published:** 1898-09-10

**Authors:** 


### Progress in Medicine.

### DISEASES OF THE LUNGS.

(*Continued from page* 372.)

**Pleurisy.**—Pitres[6] has recently made important observations on the area of dulness on percussion in pleural effusion, with special reference to its limits and the alterations of these which occur on changing the patient's position.

The examination is made, first by percussing the patient's chest whilst he is lying on his back and marking the limits with a dermographic pencil. The back is next percussed, the patient sitting up, and lastly the front of the chest, the patient continuing to sit up, the limits of dulness being marked out each time. Variations in the height and form of the dulness resulting from change of position, if present, constitute a sign of the greatest value, and form an almost absolute proof of the presence of fluid.

Pitres finds: (1) That in small effusions, the patient being seated, the dulness is found at the lateral aspect of the chest in the axillary line, with an almost horizontal upper and a curved lower border. In these small effusions the lateral dulness quite disappears when the patient lies on his back. (2) In effusions of moderate amount, the patient being seated, the upper limit of dulness in front and laterally is a horizontal line. Posteriorly, at a distance of from two to three inches from the vertebral border, the line of dulness abruptly falls, forming a triangle in which resonance persists below the general level of dulness. In some cases also there is above the level of dulness an intermediate zone of subresonance, probably due to pulmonary collapse. In most of these cases of moderate effusion there is a change in the form of the dulness with change of position by the patient. Usually the anterior level of dulness becomes raised when the patient sits up, but sometimes the reverse occurs, and it becomes lower. When there are large effusions there is no alteration in the shape of the dull area with change of position.

In about a third of the cases the areas of dulness assume abnormal forms, depending on various circumstances; for instance, it may be suspended in the upper part of the thorax, as the result of adhesions in the lower part.

**Intrapleural Tension.**—West[7] points out that the two layers of pleura which in health are in close contact, are subject to a constant strain which tends to pull them apart, called the "intrapleural" tension, which is for all practical purposes equal in amount to the elastic contraction of the lung, but opposite in direction. The respiratory movements cause constant variations in this tension, owing to the different degree to which the lungs are on the stretch in the different phases of respiration. Besides this the air is somewhat hampered both in its entrance and exit from the air tubes to the extent of half a millimetre of mercury on inspiration, and a half to two millimetres in expiration. During normal quiet breathing, both on inspiration and expiration, the intrapleural tension is negative. During forced inspiration the negative value is increased, but during forced expiration the negative value may be converted into a positive one; but as long as the two layers of pleura are in contact this positive pressure is supported by the chest walls and does not affect the pleura. When one side of the chest is opened, the admission of air goes a long way to satisfy the elasticity of the lungs on both sides. When the opening in the chest walls is at least as large as the trachea air will pass in and out easily during respiration, and, no effect being produced on the lung, it will collapse by virtue of its own elasticity, yet not completely. On the contrary, the lung is frequently found considerably expanded, and quite near the chest walls, especially if there be forced expiratory efforts, as in coughing. Where the opening is a small one the pressure will rise during expiration, and tend to compress the lung, and this is almost always the case where the perforation is from within, *e.g.*, from the lung, as in cases of pneumothorax. During inspiration in pneumothorax the air will enter the pleura as long as the pressure within the pleura is below that of the atmosphere; the inspiratory pressure cannot therefore exceed that of the atmosphere. The expiratory pressure in pneumothorax is always positive, and may be high if expiration is forcible. Thus considerable pressure will be exercised on the lung and mediastinum, so that the lung will be compressed and the mediastinum considerably displaced. In confirmation of this, it was found that out of 20 cases of paracentesis in pneumothorax the pressure was positive in 18.

The pleura is normally kept dry by the action of the "lymphatic pump," viz., the stomata and the valved lymphatics into which they lead, worked by the respiratory movements. Fluid may therefore accumulate in two ways—either it may be poured out in larger quantities than the pump can remove, or the amount of fluid may not be above the normal, but the pump defective. Correspondingly observations show that serous effusions vary greatly and irregularly, and that there is no definite relation between the size of an effusion and the amount of pressure. In serous effusions the respiratory oscillation is generally reduced and may be absent; and, as explained above, it is this which determines the working of the lymphatic pump. In performing paracentesis it can often be noticed that the respiratory oscillation absent at its commencement returns towards the end, *i.e.*, the lymphatic pump has been set to work once more. This explains why the removal of a small amount of fluid often determines the rapid disappearance of the rest.

[6] Pitres, Archives Cliniques de Bordeaux, Dec., 1897. [7] West, Lancet, May 14, 1898.

### DISEASES OF DIGESTIVE ORGANS.

**Xerostomia.**—The condition of "dry mouth" was first noticed by Jon. Hutchinson and Hadden in 1888. T. Harris[1] reports a case in a woman of 30 which had lasted for three years, with arrest of salivary and buccal glandular secretion, and with an enlargement of the parotid glands, the latter being rarely associated with the affection, and usually due to some nerve lesion. The cause is unknown, but the writer refers to more than twelve recorded cases of dry mouth, chiefly in elderly women. In his patient taste and smell were almost entirely absent, and the teeth had fallen out. A. J. Sharp[2] describes another patient, a woman, aged 41, who had suffered for 18 months. The teeth were good, and the parotid gland uninflamed. It is useful to refer here to a paper of Oslers[3] on chronic symmetrical enlargement of the salivary glands without dry mouth. He describes a case associated with syphilis and tuberculosis, and refers to Kümmel's observations on several others.

**Ulcer of the Stomach.**—Lyon[4] advocates a milk diet, with rest in bed, and, when hæmorrhage or much vomiting occurs, rectal feeding. He gives 10 to 15 grammes of bismuth, or a solution of nitrate of silver, and for the hyperacidity hot compresses and saline draughts. Large doses of magnesia or bicarbonate of soda are given for pain, and for intractable cases resort may be had to gastro-enterostomy. Hartman[5] denies the therapeutic effect of gastro-enterostomy, and quotes statistics showing eight deaths out of twelve cases, due to uncontrollable hæmatemesis. The operation may be of value in the early stages of ulceration, but not when the disease is advanced, though useful in grave, intractable cases of dyspepsia from other causes. Seymour Taylor,[6] at the Medical Society of London, argued that chronic and acute ulcers are totally different diseases. The latter he regarded as a neurosis associated with chlorosis, and akin to the perforating ulcers of tabes or those seen in herpes. Other theories of their causation are involved in hopeless difficulties, and he noted the absence of all signs of inflammation around the ulcers, their uniform shape, and their occurrence almost universally in chlorotic women. The chronic ulcer of elderly men is irregular in shape, and surrounded by thickened edges. Theodore Williams, Maguire, and others acknowledged the frequent concurrence of acute ulcers and chlorosis, but thought that it was unnecessary to invoke a neurosis, since in states of low vitality all ulcers are hard to heal. G. Reid and Purkiss[7] have both advocated perchloride of iron in the treatment of hæmatemesis, and Hale White reports the use of half drachm or drachm doses of the liquor ferri perchloridi with an equal quantity of glycerine, to be swallowed every hour or two until the bleeding stops. It was pointed out in a recent number of The Hospital, however, that iron tends to increase the hyperacidity of disordered stomachs, so that this remedy must be used with caution. [Calcium chloride is well borne in gastric ulcer, and the writer of this abstract has frequently given it with or without the carbonate of bismuth with the best results.] F. S. Hone,[8] of South Australia, discusses a rare complication, viz., parotiditis, of which he reports a case in detail, and refers to eight others. The affection may be unilateral or bilateral, may occur a few days or a fortnight after hæmatemesis, and may resolve or end in suppuration. He criticises a theory in Allbutt's System which ascribes it to the absence of saliva produced by rectal feeding, but he finds that it arises even when the patient is fed by the mouth, and it is not recorded in other diseases where rectal feeding is employed. Paget gives a long list of gastro-intestinal injuries and diseases where it has been found, but in only 15 out of 101 was there general pyæmia. Hone does not regard as a satisfactory explanation of this complication either the septic theory, or that of a reflex neurosis, or that a dry, septic state of the mouth leads to infection along Steno's duct. If the last theory is sound, and if the swelling of the parotid in xerostomia is analogous, why does the submaxillary gland almost always escape in gastro-intestinal cases?

**Cancer, Syphilis, and Tuberculosis of the Stomach.**—McRae analyses 150 cases of cancer from the John Hopkins Hospital.[9] The majority of the patients were between 40 and 60 years of age. The diagnosis was generally made post-mortem. Ten made no complaint of stomach trouble at all, in 39 the onset was sudden, while in 46 there had been illness for three months and over. Friedenwald, in a paper on latent cancer, remarked that latency occurred when the growth was not situated near an orifice, and when the surface was not ulcerated. True tuberculous ulcers, containing bacilli and of typical structure, were found by Blumer[10] in the stomach of a patient suffering from miliary tuberculosis. He could only discover 30 well authenticated instances of this condition. Probably the acid, when the mucosa is of low vitality, is unable to protect the latter; but direct ingrowth from the peritoneum, or an interference with the vascular supply has been the cause of some of these ulcers. Dieulafoy[11] mentions an instance where an intractable ulcer in an old syphilitic was cured by active specific treatment, and remarks that these affections are not so rare as is supposed. Indeed, the syphilitic lesions of the stomach are various, and their condition is made worse by the gastric juice. There are no symptoms differing from those of simple ulcer, but specific treatment is necessary. Flexner[12] finds that syphilis of the stomach may be hereditary or acquired, and describes a case of perforated specific ulcer. Death occurred soon after the man had eaten a hearty meal. The diagnosis was based on microscopical examination. The liver also contained a large gumma.

[1] Amer. Jour. Med. Sci., Mar. [2] Lancet, April 3. [3] Amer. Jour. Med. Sci., Jan. [4] La Semaine Med., Mar. 30. [5] Med. Rec., May 7. [6] Lancet, Mar. 19. [7] Brit. Med. Jour., July 9. [8] Australas. Med. Jour., Feb. [9] Med. Rec., May 21. [10] Brit. Med. Jour., June 18. [11] La Semaine Med., May 18. [12] Med. Rec., May 14.

